# Jagged-2 (JAG2) enhances tumorigenicity and chemoresistance of colorectal cancer cells

**DOI:** 10.18632/oncotarget.18391

**Published:** 2017-06-07

**Authors:** Vivek Vaish, Joohwee Kim, Minsub Shim

**Affiliations:** ^1^ Department of Biological Sciences, University of South Carolina, Columbia, SC, 29208 USA; ^2^ Center for Colon Cancer Research, University of South Carolina, Columbia, SC, 29208 USA; ^3^ Present address: Assistant Professor, Department of Biotechnology, Savitribai Phule Pune University, Pune 411 007 Maharashtra, India

**Keywords:** chemoresistance, doxorubicin, Jagged-2, NOTCH, p21

## Abstract

Colorectal cancer (CRC) is one of the leading causes of cancer-related mortality. Recent studies have stated that NOTCH signaling plays an important role in the development and progression of CRC. However, the role of Jagged-2 (JAG2), one of the NOTCH ligands, has not been delineated in colorectal tumorigenesis and drug resistance. In the present study, we have examined the impact of targeting JAG2 on CRC cells. Among all the members of NOTCH ligands, only the expression of JAG2 was found up-regulated in the intestinal tumors of Apc*^Min^*/+ mice as compared to the nearby normal mucosa. JAG2 expression was also observed in a panel of human CRC cell lines. Pharmacological inhibition or genetic knockdown of β-catenin in CRC cell lines suppressed JAG2 expression, suggesting Wnt/β-catenin regulation of JAG2 expression. In addition, deletion of Apc gene in the intestinal cells of Apc conditional knockout mice resulted in up-regulation of JAG2 expression. Modulation of JAG2 expression significantly affected *in vivo* tumorigenicity of CRC cell lines. Moreover, knockdown of JAG2 sensitized CRC cells to chemotherapeutic agents, while ectopic expression of JAG2 increased chemoresistance of the CRC cells. Significant down-regulation of p21 was observed in JAG2-knockdown cells. Forced expression of p21 rescued the sensitivity of JAG2-knockdown cells to doxorubicin. In addition, the chemosensitivity of p21-null cells was not affected by JAG2 knockdown. These results suggest that JAG2 modulates the sensitivity of CRC cells to chemotherapeutic agents through p21. Our study identifies JAG2 as a novel target for therapeutic intervention of CRC.

## INTRODUCTION

Colorectal cancer (CRC) has become a major cause of cancer-related deaths among the Western countries. Mutations (both hereditary and somatic) that contribute to adenoma formation or progression have been identified in a number of genes [1]. Most significantly, mutations in genes involved in Wnt signaling, such as the adenomatous polyposis coli (APC) gene, cause colon cancer through the constitutive formation of a nuclear β-catenin/TCF transcription complex in colonic epithelial cells [2]. Finally, studies on the basic mechanisms of cell fate specification and differentiation of stem and progenitor cells in the gastrointestinal tract led to the discovery that NOTCH activation is essential for adenoma formation in Apc*^Min^*/+ mice, a well-established model for intestinal tumorigenesis [3].

The NOTCH pathway is an evolutionarily conserved signaling system involved in regulation of cell fate during embryonic development [4]. In mammalian cells, NOTCH signaling consists of four receptors (NOTCH1-NOTCH4), three delta-like ligands (DLL1, DLL3, and DLL4), and two serrate-like ligands (JAG1 and JAG2) [5]. NOTCH activation is mediated by a direct contact between cells expressing NOTCH ligands and receptors. The interaction between a receptor and a ligand results in activation of the γ-secretase protein complex, resulting in cleavage of the NOTCH receptor and subsequent release of the NOTCH intra-cellular domain (NICD). The released NICD translocates to the nucleus and binds to transcription factors such as RBP-Jκ to form a transcription-activating complex. The formation of this complex induces expression of downstream target genes such as HES1.

All four NOTCH receptors and five ligands have been shown to be expressed in fetal and adult mouse intestine [6, 7]. In the human colon, NOTCH1, NOTCH2, and NOTCH3 are expressed at the basal crypt, while JAG1 is present at the top of the crypts [8]. Consistent with its central role in cell-cell communication and cell-fate determination, aberrant activation of NOTCH signaling is frequently observed in many human cancers including CRC [9, 10]. Additionally, NOTCH signaling increases chemoresistance by protecting tumor cells from apoptosis [11–14]. We recently reported a novel mouse model of sporadic CRC using conditional Apc knockout (Apc^CKO^) mice, in which intra-rectal administration of Cre-encoding lentivirus resulted in stochastic transduction of colonic epithelium, leading to the inactivation of floxed Apc gene and subsequent tumor development [15]. High levels of NICD and HES1 expression were observed in the developed tumors, suggesting activation of NOTCH signaling. In addition, a high-level expression of JAG2, one of the NOTCH ligands, was also observed in the colorectal tumors of Apc^CKO^ mice, suggesting the role of JAG2 in colorectal tumorigenesis.

JAG2 over-expression was first identified in malignant plasma cells from patients with multiple myeloma [16]. Up-regulation of JAG2 expression was also observed in various cancers, including breast, pancreatic, bladder, and lung cancers, and was associated with the progression of these tumors [17–20]. Reedijk et al. [21] reported that JAG2 expression is increased in human CRC compared to that in the surrounding normal tissues, suggesting that dysregulation of JAG2 expression plays a role in CRC cell growth and progression to metastatic disease. In addition, our search of the Oncomine database (www.oncomine.org) [22] revealed that JAG2 expression is up-regulated in human colorectal carcinoma compared to that in normal epithelium [23–25]. Although the function of other NOTCH ligands, such as JAG1, in CRC has been examined [26–28], the precise role of JAG2 in CRC remains unclear.

In the present study, we found that JAG2 expression is regulated by the aberrant Wnt/β-catenin signaling. We hypothesized that JAG2 functions as a downstream mediator of Wnt/β-catenin signaling and, thus, that targeting of JAG2 could generate growth arresting effects in CRC cells. We found that silencing of JAG2 suppresses the growth of CRC xenografts, while its over-expression increases tumor growth. We also found that JAG2 plays an important role in chemoresistance of CRC cells through modulation of p21.

## RESULTS

### JAG2 expression is increased in tumors of Apc^*Min*/+^ mice and human CRC cell lines

**Figure 1:**
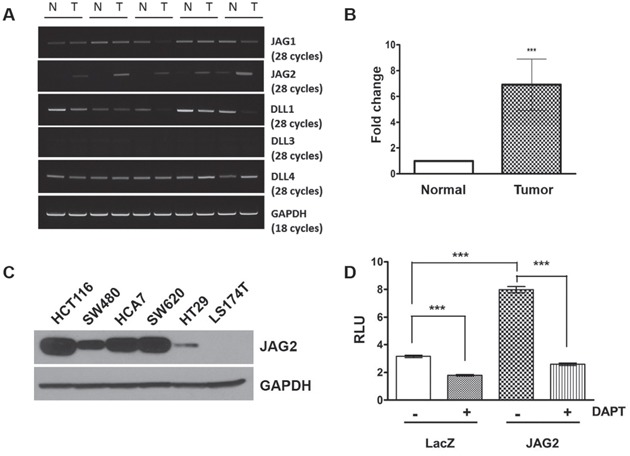
JAG2 expression in intestinal tumors of Apc^*Min*/+^ mice and human CRC cell lines **(A)** RT-PCR analysis for NOTCH ligands in the normal intestine (N) and tumor (T) samples of APC^*Min*/+^ mice. GAPDH served as a loading control. **(B)** Quantitative real-time PCR (qRT-PCR) analysis for JAG2 expression in normal and tumor samples of APC^*Min*/+^ mice. JAG2 expression in 5 tumor samples was calculated as mean fold change relative to surrounding normal tissue using the 2^−ΔΔC^_T_ method (p<0.001). **(C)** Western blot analysis for JAG2 in human CRC cell lines. X-ray film was exposed for a longer duration to detect JAG2 expression in all the cell lines tested. **(D)** HCT116 cells were co-transfected with NOTCH reporter plasmid (pHes1-luc), pRL-null plasmid, and empty vector or JAG2 expression plasmid. Twenty-four hours after transfection, the cells were treated with 10 μM DAPT for 24 h. Notch reporter activity was normalized to Renilla luciferase activity. These experiments are the representative images of more than 3 independent experimentations.

We recently reported that JAG2 expression is up-regulated in colorectal tumors of Apc^CKO^ mice resulting from transduction of colonic epithelium with Cre-encoding lentivirus [15]. To determine JAG2 expression in other mouse models of CRC, we analyzed JAG2 expression in the intestinal tumors of Apc^*Min*/+^ mice. RT-PCR analysis showed that JAG2 expression was consistently higher in the tumors of Apc^*Min*/+^ mice where NOTCH signaling has been shown to be active [3], compared to that in corresponding normal mucosa, whereas the expression of other NOTCH ligands was variable (Figure 1A). Quantitative RT-PCR analysis revealed that the average expression of JAG2 mRNA in tumor tissues was ∼7 fold higher as compared to normal epithelium while the mRNA expression of other NOTCH ligands was not significantly different between tumor and normal epithelium (Figure 1B and Supplementary Figure 1A). Immunohistochemical analysis revealed that the expression of JAG2 and HES1, a downstream target of NOTCH signaling, was increased in the intestinal tumors of Apc^*Min*/+^ mice (Supplementary Figure 1B and 1C). We also examined JAG2 levels in a panel of human CRC cell lines. After a long exposure of the Western blot, expression of the JAG2 protein was detectable in most of the human CRC cell lines examined (Figure 1C). JAG2 expression was not detectable in LS174T cell line. To determine whether JAG2 contributes to activation of NOTCH signaling, HCT116 cells were transfected with the NOTCH reporter and JAG2 expression plasmids. This resulted in the enhanced transcriptional activity of the NOTCH reporter (Figure 1D). JAG2-induced increase in reporter activity was suppressed by DAPT, a γ-secretase inhibitor, suggesting that the expression of JAG2 may contribute to the activation of NOTCH signaling in intestinal tumors.

### β-catenin signaling is involved in regulation of JAG2 expression

**Figure 2:**
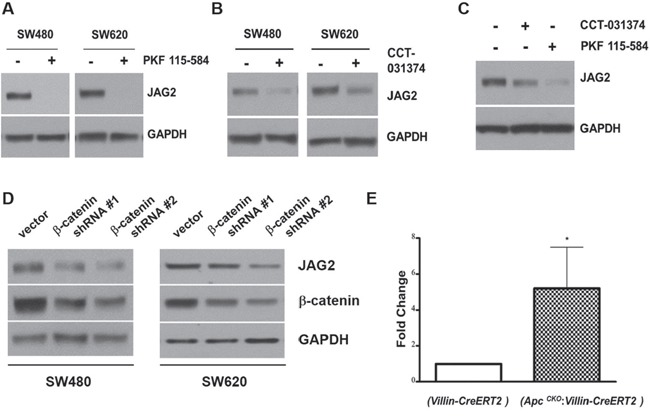
β-catenin regulates JAG2 expression SW480 and SW620 cells were incubated with 1 μM PKF 115-584 **(A)** or 25 μM CCT-031374 **(B)** for 24 hours. Total cell lysates were subjected to Western blot analysis for JAG2. **(C)** HCT116 cells were incubated with 1 μM PKF 115-584 or 25 μM CCT-031374 for 24 hours. JAG2 expression was analyzed by Western blot analysis. **(D)** SW480 and SW620 cells were transduced with lentiviral shRNAs against β-catenin. 72 hours after transduction, total cell lysates were subjected to Western blot analysis for JAG2 and β-catenin. All Western blot images are representative of at least three independent experiments. **(E)** qRT-PCR analysis of JAG2 mRNA levels in control (Villin-CreERT^2^) and conditional Apc knockout (Apc^CKO^:Villin-CreERT^2^) mice. The mice were intra-peritoneally injected with tamoxifen. RNA was isolated from small intestines of the injected mice at 4 days after Cre activation. The relative expression level of JAG2 mRNA was normalized to GAPDH. Data were expressed as fold change over tamoxifen-injected control mice (Villin-CreERT^2^, n=3, * for p < 0.05).

Aberrant activation of Wnt/β-catenin signaling due to the mutations in Apc gene is common in CRC. Since JAG2 expression was increased in mouse models of CRC and human CRC cell lines, we wondered whether Wnt/β-catenin signaling is involved in JAG2 expression. To test this, SW480 and SW620 cells were incubated with PKF115-584, an inhibitor of β-catenin signaling. PKF115-584 has been reported to interfere with the formation of Tcf/β-catenin complex as well as with the binding of Tcf/β-catenin complex to DNA [29]. SW480 and SW620 cell lines exhibit a relatively high level of JAG2 expression, thus allowing the analysis of JAG2 levels in response to inhibition of Wnt/β-catenin signaling. As shown in Figure 2A, PKF115-584 abrogated JAG2 expression in these cell lines. To validate this result, we treated the cells with CCT-031374, another inhibitor of β-catenin signaling. As shown in Figure 2B and Supplementary Figure 2A, treatment with CCT-031374, which causes destabilization of the β-catenin protein [30], also reduced JAG2 expression in SW480 and SW620 cells. PKF 115-584 or CCT-031374 treatment also reduced JAG2 expression in HCT116 cells (Figure 2C and Supplementary Figure 2B). Both inhibitors also significantly reduced JAG2 mRNA levels in SW480 and HCT116 cell lines, suggesting that β-catenin may regulate JAG2 mRNA expression (Supplementary Figure 2C). To confirm that β-catenin signaling is involved in regulation of JAG2 expression, we knocked down β-catenin expression using lentivirus. Since stable knockdown of β-catenin resulted in growth inhibition and cell death (data not shown), the cells were harvested at 72 hours after transduction. As shown in Figure 2D and Supplementary Figure 2D, knockdown of β-catenin significantly reduced JAG2 expression in SW480 and SW620 CRC cell lines. To determine β-catenin regulation of JAG2 expression *in vivo*, we analyzed JAG2 mRNA expression in the small intestine of Apc^CKO^ mice, which have floxed Apc alleles. Apc^CKO^ mice were crossed to Villin-CreERT^2^ mice, which expresses tamoxifen-inducible Cre recombinase in the intestinal epithelial cells, to generate Apc^CKO^:Villin-CreERT^2^ mouse. The resulting Apc^CKO^:Villin-CreERT^2^ mice were intra-peritoneally injected with tamoxifen to induce deletion of Apc gene (Supplementary Figure 2E) and sacrificed 4 days later. Consistent with the results of Peignon et al. [29], JAG2 mRNA expression was significantly up-regulated upon deletion of Apc alleles (Figure 2E). Immunohistochemical localization of JAG2 in small intestines revealed significant up-regulation of JAG2 in the Apc^CKO^:Villin-CreERT^2^ mice treated with tamoxifen as compared to the Villin-CreERT^2^ mice (Supplementary Figure 2F). Consistent with previous reports from Villin-CreERT^2^ mice which exhibit higher expression of Cre in the epithelial cells of the villi [30, 31], staining of JAG2 was stronger in the differentiated cells of villi as compared to the the crypt cells.

### Modulation of JAG2 affects tumorigenicity of CRC cells

**Figure 3:**
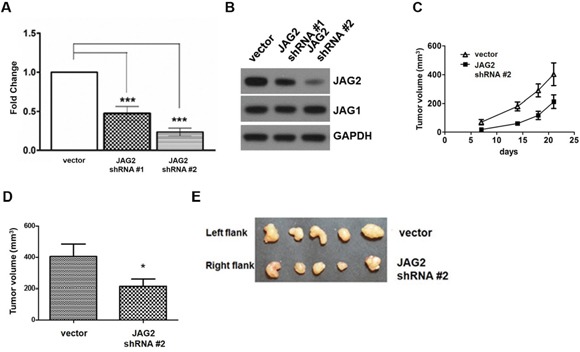
Knockdown of JAG2 reduces tumorigenicity **(A)** qRT-PCR analysis of JAG2. The relative JAG2 expression in HCT116 cells transduced with the empty vector- or JAG2 shRNA-encoding lentivirus normalized to GAPDH. **(B)** Western blot analysis of JAG2 and JAG1 in HCT116 cells transduced with empty vector or JAG2 shRNA-encoding lentivirus. The Western blot is representative of three independent experiments (n=3). **(C)** The tumor growth curve. 1x 10^6^ empty vector (vector) or JAG2-knockdown HCT116 cells were injected into left and right hind legs of each mouse, respectively. Tumor volumes were measured at 7, 14, and 18 days and tumor tissues were harvested at 21 days after cell injection. The average tumor volume **(D)** at the time of harvest and harvested tumor tissues **(E)** are shown (n=5).

To analyze the role of JAG2 in colorectal tumorigenesis, HCT116 cells were transduced with the lentiviruses expressing shRNAs against JAG2, and the resulting puromycin-resistant clones were pooled. As shown in Figure 3A, quantitative RT- PCR analysis revealed that the expression of JAG2 shRNA resulted in 55% and 70% reduction of JAG2 mRNA, respectively, compared to that of the control cell line. The reduced expression of JAG2 protein was also confirmed by Western blot analysis (Figure 3B and Supplementary Figure 3A). Stable expression of JAG2 shRNA did not affect JAG1 levels. Control and JAG2-knockdown HCT116 cells were then subcutaneously injected into the flanks of nude mice. As shown in Figure 3C, knockdown of JAG2 decreased the rate of tumor growth. At harvest, the average volume of tumors from JAG2-knockdown cells was significantly reduced than those of tumors from control cells (Figure 3D and 3E). The low expression of JAG2 and HES1 in tumors of JAG2-knockdown HCT116 cells was validated by immunohistochemical analysis (Supplementary Figure 3B and 3C).

**Figure 4:**
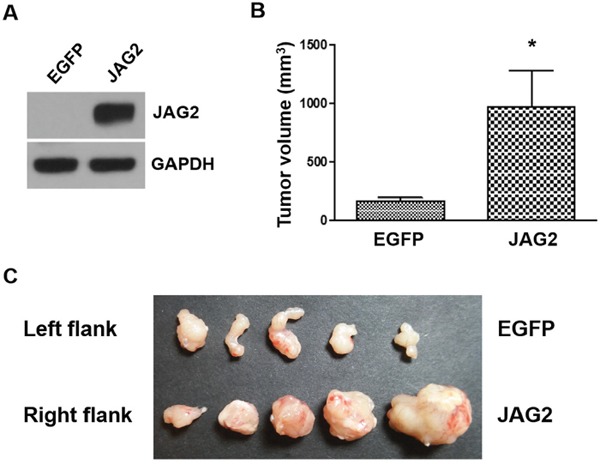
Over-expression of JAG2 enhances tumorigenicity **(A)** Western blot analysis of JAG2 in MC38 cells transduced with EGFP or JAG2-encoding lentivirus. 1x 10^6^ control (EGFP) or JAG2 over-expressing MC38 cells were injected into left and right hind legs of each mouse, respectively. The average tumor volume **(B)** at 21 days after injection and harvested tumor tissues **(C)** are shown (n=5).

To further analyze the role of JAG2 in the tumorigenicity of CRC cells, we generated MC38 cells, a cell line derived from mouse colon adenocarcinoma [32], that constitutively express JAG2 using lentivirus (Figure 4A). The human and mouse JAG2 are 90% identical as compared using the protein BLAST. Endogenous JAG2 expression was not detectable in control MC38 cells (Supplementary Figure 4A). Control and JAG2 over-expressing MC38 cells were subcutaneously injected into the flanks of syngeneic C57BL/6 mice. As shown in Figure 4B and 4C, ectopic expression of JAG2 resulted in a significant increase in tumor size, suggesting that JAG2 enhances tumorigenicity of MC38 cells. The expression of HES1 was increased in the tumors of JAG2 over-expressing MC38 cells (Supplementary Figure 4B and 4C).

### Silencing of JAG2, but not JAG1, sensitizes HCT116 cells to chemotherapy

**Figure 5:**
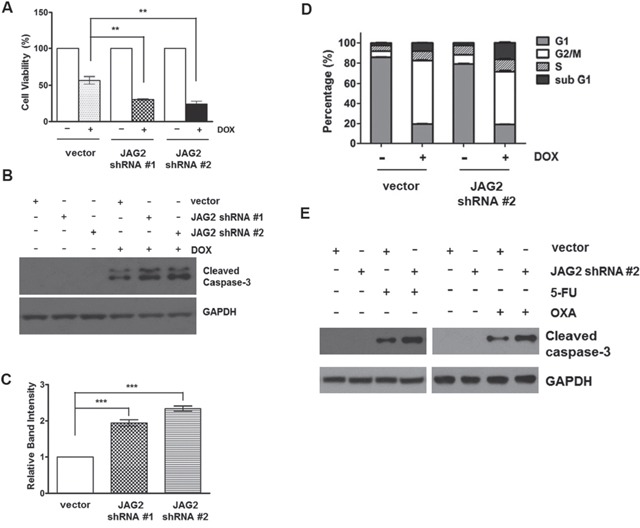
Silencing of JAG2 sensitizes HCT116 cells to doxorubicin and other chemotherapeutic agents **(A)** Control and JAG2-knockdown HCT116 cells were treated with 1 μM doxorubicin (DOX) for 48 hours. Cell viability was determined by trypan blue assay and expressed as a percentage of vehicle-treated cells. Bar graphs show mean ± SD of percent decrease of viable cells over DMSO-treated control (100%). **(B)** Control and JAG2-knockdown HCT116 cells were treated with 1 μM DOX for 48 hours. Total cell lysates were subjected to Western blot analysis for cleaved caspase-3. The Western blot is representative of 4 independent experiments (n=4). **(C)** Quantification of GAPDH-normalized cleaved caspase-3 over DOX-treated control cells. **(D)** Control and JAG2-knockdown HCT116 cells were treated with 1 μM DOX for 48 hours, and then harvested for analysis of cell cycle distribution using flow cytometry. The bar graph represents the distribution of the cells in different phases of the cell cycle. (n=3) **(E)** HCT116 cells were treated with 100 μM 5-fluorouracil (5-FU) or 10 μM oxaliplatin for 48 hours. Total cell lysates were subjected to Western blot analysis for cleaved caspase-3 and GAPDH. Representative blot of 3 independent experiments is shown (n=3).

To investigate the potential role of JAG2 in the modulation of CRC cell survival, JAG2-knockdown HCT116 cells were treated with doxorubicin (DOX), and the viability of the cells was analyzed. As shown in Figure 5A, knockdown of JAG2 expression increased cytotoxic response to DOX. This increase in cytotoxicity is likely to result from increased apoptosis as the levels of the cleaved caspase-3 were significantly increased in JAG2-knockdown cells (Figure 5B and 5C). The sensitivity to DOX was slightly higher in HCT116 cells stably transduced with JAG2 shRNA #2 than in those transduced with JAG2 shRNA #1, possibly due to the higher knockdown efficiency of JAG2 shRNA #2 (Figure 3A and 3B). In addition, flow cytometry analysis with these cell lines revealed that the percentage of apoptotic sub-G1 cells was increased in DOX-treated JAG2-knockdown cells compared to that in DOX-treated control cells (Figure 5D). In addition, as shown in Figure 5E, knockdown of JAG2 further increased the levels of cleaved caspase-3 following treatment with other chemotherapeutic agents, including 5-fluorouracil (5-FU) and oxaliplatin (OXA).

**Figure 6:**
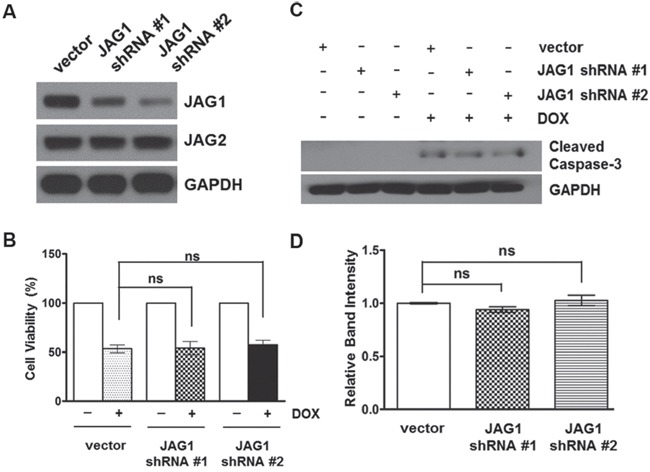
Silencing of JAG1 does not affect the sensitivity of HCT116 cells to doxorubicin **(A)** Western blot analysis of JAG1 and JAG2 in HCT116 cells transduced with empty vector or JAG1 shRNA-encoding lentivirus. **(B)** Control and JAG1-knockdown HCT116 cells were treated with 1 μM doxorubicin (DOX) for 48 hours. Cell viability was determined by trypan blue assay and expressed as a percentage of vehicle-treated cells (n=3)., ns: non-significant. **(C)** Control and JAG1-knockdown HCT116 cells were treated with 1 μM DOX for 48 hours. Total cell lysates were subjected to Western blot analysis for cleaved caspase-3. The Western blot is representative of 3 independent experiments (n=3). **(D)** Quantification of cleaved caspase-3. The graph shows fold changes of GAPDH-normalized cleaved caspase-3 over DOX-treated control cells.

To examine the specificity of JAG2 knockdown on chemosensitivity of CRC cells, we stably knocked down JAG1, the other serrate-like NOTCH ligand (Figure 6A and Supplementary Figure 5). However, knockdown of JAG1 did not affect the cytotoxic response to DOX (Figure 6B). Moreover, the levels of cleaved caspase-3 were not significantly different between DOX-treated control and JAG1-knockdown HCT116 cell lines (Figure 6C and 6D). Stable expression of JAG1 shRNA did not affect JAG2 protein levels (Figure 6A and Supplementary Figure 5).

### Silencing of JAG2 sensitizes p53-null HCT116 cells to DOX

**Figure 7:**
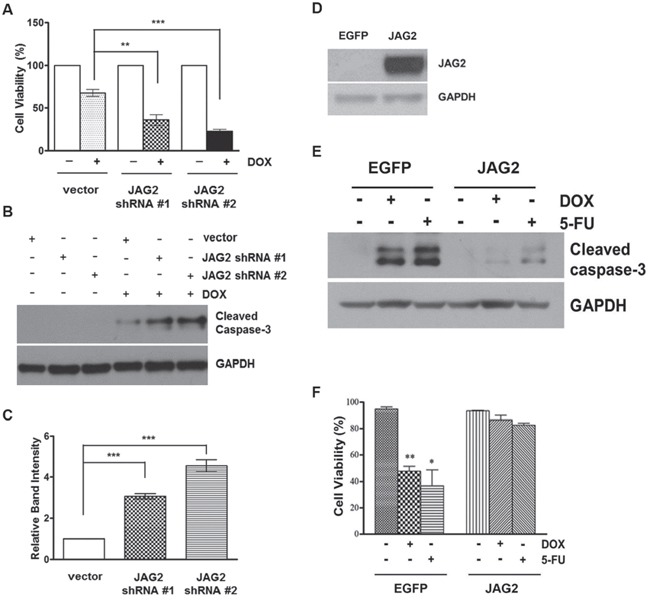
Knockdown of JAG2 sensitizes p53-null HCT116 cells to doxorubicin **(A)** Control and JAG2-knockdown p53-null HCT116 cells were treated with 1 μM doxorubicin (DOX) for 48 hours. Cell viability was determined by trypan blue assay and expressed as a percentage of vehicle-treated cells. Bar graphs show mean ± SD of percent decrease of viable cells over DMSO-treated control (100%). **(B)** Control and JAG2-knockdown p53-null HCT116 cells were treated with 1 μM DOX for 48 hours. Total cell lysates were subjected to Western blot analysis for cleaved caspase-3. **(C)** The graph shows fold changes of GAPDH-normalized cleaved caspase-3 over DOX-treated control cells. **(D)** Western blot analysis of JAG2 expression in control (EGFP) and JAG2 over-expressing HT29 cells. X-ray film was exposed for a shorter duration to properly visualize JAG2 expression in JAG2 over-expressing HT-29 cells. **(E)** Control and JAG2 over-expressing HT29 cells were treated with 1 μM doxorubicin or 300 μM 5-FU for 48 hours for 24 hours. Total cell lysates were subjected to Western blot analysis for cleaved caspase-3. All Western blot images are representative images from at least 3 independent experiments. **(F)** Calcein AM/DAPI viability assay. Control and JAG2 over-expressing HT29 cells were treated with 1 μM doxorubicin or 300 μM 5-FU for 48 hours. Treated cells were stained with Calcein AM and DAPI for visualization of live and dead cells, respectively. Data are presented as the percent of live cells.

Many chemotherapeutic agents act by causing either direct or indirect damage to cellular DNA. One of the critical components of DNA damage response is the tumor suppressor protein p53. To investigate the mechanism by which JAG2 modulates chemosensitivity, p53-null HCT116 cells were transduced with JAG2 shRNA-encoding lentiviruses, and the resulting puromycin-resistant pools were treated with DOX. As shown in Figure 7A, knockdown of JAG2 also sensitized p53-null HCT116 cells to DOX. Similar to the results with p53 wild-type HCT116 cells, DOX-induced expression of cleaved caspase-3 was significantly increased in JAG2-knockdown p53-null HCT116 cells compared to that in control p53-null cells (Figure 7B and 7C). These results suggest that sensitization of HCT116 cells to DOX through silencing of JAG2 occurs in a p53-independent manner. In line with this notion, we also found that over-expression of JAG2 (Figure 7D) increases chemoresistance in HT29 cells carrying mutant p53. As shown in Figure 7E, significantly reduced levels of cleaved caspase-3 were observed when JAG2 over-expressing HT29 cells were treated with DOX or 5-FU. This effect of JAG2 expression on the enhancement of chemoresistance was further verified by Calcein-AM/DAPI staining for viable/dead cells (Figure 7F). A significant amount of cell death was observed in control HT29 cells treated with DOX or 5-FU. However, JAG2 over-expressing HT29 cells were observed to be resistant towards cell death after the treatment of DOX and 5-FU.

### Sensitization of HCT116 cells to DOX by JAG2-silencing requires p21

**Figure 8:**
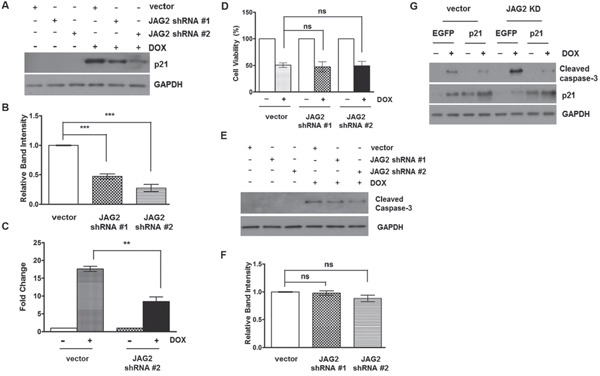
Knockdown of JAG2 sensitizes HCT116 cells to doxorubicin in a p21-dependent manner **(A)** Control and JAG2- knockdown HCT116 cells were treated with 1 μM doxorubicin (DOX) for 48 hours. Total cell lysates were subjected to Western blot analysis for p21. The graph below **(B)** shows fold changes of GAPDH-normalized p21 over DOX-treated control cells. **(C)** qRT-PCR analysis of p21 mRNA levels in DOX-treated control and JAG2-knockdown HCT116 cells. The relative expression level of p21 was normalized to GAPDH. The data are the mean ± SD of three independent experiments and are expressed as fold induction over vehicle-treated cells. **(D)** Control and JAG2-knockdown p21-null HCT116 cells were treated with 1 μM DOX for 48 hours. Cell viability was determined by trypan blue assay and expressed as a percentage of vehicle-treated cells. Bar graphs show mean ± SD of percent decrease of viable cells over DMSO-treated control (100%)., (n=3) **(E)** Control and JAG2-knockdown p21-null HCT116 cells were treated with 1 μM DOX for 48 hours. Total cell lysates were subjected to Western blot analysis for cleaved caspase-3. The graph below **(F)** shows fold changes of GAPDH-normalized cleaved caspase-3 over DOX-treated control cells. **(G)** Control and JAG2-knockdown wild-type HCT116 cells were transduced with EGFP- or human p21-encoding lentiviruses and then treated with 1 μM DOX for 48 hours. Total cell lysates were subjected to Western blot analysis for cleaved caspase-3, p21, and GAPDH. All Western blot images are representative images from at least 3 independent experiments.

As shown in Figure 5D, DOX treatment arrested the control HCT116 cells in G2/M, the phase where most of the cells had been shown to be arrested by DOX through its effect on topoisomerase II [33]. However, knockdown of JAG2 reduced DOX-induced G2/M arrest, suggesting that defects in G2 arrest due to JAG2 knockdown may lead to increased apoptosis. p21 is known to induce G2 arrest and protect the cells against apoptosis through its ability to promote cell cycle inhibition [34]. To investigate the possible role of p21 in increased sensitivity of JAG2-knockdown cells to DOX, we assessed p21 protein levels between control and JAG2-knockdown p53 wild-type HCT116 cells. As shown in Figure 8A and 8B, p21 protein expression was highly induced by DOX in control cells. However, DOX-induced p21 expression was greatly reduced in JAG2-knockdown cells. Knockdown of JAG1 did not affect DOX-induced p21 levels (Supplementary Figure 6). qRT-PCR analysis revealed that induction of p21 mRNA was significantly suppressed in DOX-treated JAG2-knockdown cells compared to that in DOX-treated control cells (Figure 8C). Similarly, DOX-induced p21 expression was also decreased in p53-null JAG2-knockdown HCT116 cells (Supplementary Figure 7).

To determine whether p21 is involved in increased sensitivity of JAG2-knockdown cells to DOX, we stably knocked down JAG2 in p21-null HCT116 cells. As shown in Figure 8D, DOX-induced cytotoxicity was not significantly altered between control and JAG2-knockdown p21-null HCT116 cells. Moreover, the levels of cleaved caspase-3 were not significantly different between control and JAG2-knockdown p21-null HCT116 cells (Figure 8E and 8F). To provide further evidence that reduced p21 expression is responsible for increased sensitivity of JAG2-knockdown cells to DOX, we examined whether forced expression of p21 can rescue the increased apoptosis in JAG2-knockdown cells. To test this, p53 wild-type control and JAG2-knockdown HCT116 cells were transduced with the lentivirus encoding human p21. Twenty-four hours later, the transduced cells were treated with DOX, and expression of cleaved caspase-3 was examined. As shown in Figure 8G, the level of cleaved caspase-3 was significantly up-regulated in DOX-treated JAG2-knockdown cells compared to that in DOX-treated control cells (lane 2 vs. lane 6). However, this increase was suppressed by forced expression of p21 (lane 6 vs. lane 8). Taken together, our data suggest that silencing of JAG2 interferes with DOX-induced cell cycle arrest through suppression of p21 expression, leading to increased apoptosis.

## DISCUSSION

Activation of NOTCH signaling is frequently observed in CRC. However, the mechanism that causes NOTCH activation in CRC remains to be unraveled. Constitutive activation of β-catenin signaling due to inactivating mutations in APC gene or activating mutations in β-catenin gene is a common feature of CRC. We previously have shown that JAG2 expression is increased in colorectal tumors of Apc^CKO^ mice [15]. In the current study, we also observed increased JAG2 mRNA expression in the tumors of Apc^*Min*/+^ mice, while tumoral expression of other NOTCH ligands was variable. In addition, JAG2 expression was detectable in most of the CRC cell lines examined. We have demonstrated that knockdown or pharmacological inhibition of β-catenin reduces JAG2 levels in the CRC cell lines. Moreover, acute deletion of Apc alleles in tamoxifen-treated Apc^CKO^:Villin-CreERT^2^ mice resulted in the up-regulation of JAG2 mRNA inside the small intestine. Taken together, our results suggest that aberrant activation of Wnt/β-catenin promotes JAG2 expression in CRC. Given that NOTCH signaling is activated by the ligand-receptor interaction and that mutations in NOTCH receptors are not common in CRC [35, 36], increased JAG2 expression may be responsible for aberrant NOTCH activation in CRC.

There has been increasing evidence that inhibition of NOTCH signaling suppresses CRC growth and sensitizes CRC to therapy [37, 38]. A frequent approach to blocking NOTCH activity is preventing the final cleavage step of the NOTCH receptor with γ-secretase inhibitors (GSIs), thereby inhibiting the formation of NICD. However, GSIs are not selective for different NOTCH receptors and are associated with a number of severe side effects, including gastrointestinal toxicity [39, 40]. We have demonstrated that knockdown of JAG2 sensitizes CRC cells to chemotherapeutic agents. Thus, selective targeting of JAG2 could be a potential strategy to circumvent existing therapeutic limitations of GSIs.

Rodilla et al. [27] have shown that β-catenin-mediated transcriptional activation of JAG1 is linked to NOTCH activation in CRC cell lines. Their and our results suggest that both JAG1 and JAG2 may play a role in NOTCH activation in CRC. They also reported that the deletion of single JAG1 allele resulted in reduced tumor growth in Apc^*Min*/+^ mouse model. Similarly, our data indicate that JAG2 is associated with increased tumorigenicity of CRC cell lines, suggesting that JAG1 and JAG2 may have a redundant role in CRC development. Further studies will determine the *in vivo* role of JAG2 in CRC development using tissue-specific JAG2 knockout mice, since global deletion of JAG2 is lethal [41]. Our study also suggests that JAG1 and JAG2 may have distinct roles. Although their study demonstrated the role of JAG1 in CRC development, the role of JAG1 in chemoresistance has not been investigated. We have found that knockdown of JAG2, but not of JAG1, sensitized CRC cell lines to chemotherapeutic agents. This suggests that JAG2 may have its own signaling function that is important to cell survival independent of the canonical NOTCH pathway. Alternatively, it may be possible that JAG2 may have different receptor specificity than JAG1, or elicit different responses when binding to the same NOTCH receptor. Supporting this idea, the mice that are null for the genes encoding JAG1, JAG2, or DLL4 exhibit overlapping, but clearly distinct phenotypes [5]. Interactome analysis of JAG2 would reveal the mechanism underlying JAG2-mediated chemoresistance.

We have identified p21 as a downstream effector involved in JAG2-regulation of chemoresistance. JAG2 knockdown suppressed DOX-induced expression of p21, which is an inhibitor of DNA damage-induced apoptosis [42, 43], suggesting that reduced p21 level may be responsible for the increased sensitivity of JAG2 knockdown cells to DOX. In line with this, ectopic expression of p21 rescued the sensitivity of JAG2-knockdown cells to DOX. Moreover, the sensitivity of p21-null cells to DOX was not affected by JAG2 knockdown. JAG2 appears to regulate p21 mRNA levels since knockdown of JAG2 resulted in 50% decrease in p21 mRNA induction in response to DOX treatment. JAG2 knockdown also reduced the level of p21 in DOX-treated p53-null HCT116 cells, suggesting that JAG2 regulation of p21 in these cells involves p53-independent mechanisms. It has been shown that NOTCH positively regulates p21 expression in human keratinocytes [44]. Given that JAG2 is a NOTCH ligand, it may be possible that the reduced NOTCH activity due to JAG2 knockdown contributes to decreased levels of p21. However, other mechanisms are also possible. The silencing of nuclear factor-κB (NF-κB) in p53-null HCT116 cells has been reported to enhance the cytotoxic effect of DOX through down-regulation of p21 [45]. Consistent with this, NOTCH signaling has been shown to induce IKKα-mediated NF-κB activation in human keratinocytes [46]. In addition, sphingosine kinase 2 (Sphk2) has been shown to be involved in p53-independent induction of p21 in DOX-treated HCT116 cells [47]. The level of p21 protein is also regulated by post-translational mechanisms including caspase-3-mediated cleavage [48] and proteasomal degradation [49]. However, we have found that MG-132, a proteasome inhibitor, and Z-DEVD-FMK, a caspase-3 inhibitor, did not affect p21 levels in DOX-treated JAG2-knockdown cells (Supplementary Figure 8 and data not shown).

We have made efforts to demonstrate the role of JAG2 under the stress of chemotherapeutic drugs which usually cause DNA damage as an end effect. DOX is a potent DNA damaging drug and hence was utilized. 5-FU, oxaliplatin, and irinotecan are chemotherapeutic agents frequently used in patients with CRC. Although we have shown that JAG2 knockdown sensitized the CRC cells to 5-FU and oxaliplatin, one of the limitations in our study is the lack of evidence showing *in vivo* role of JAG2 in chemoresistance. Our future study will focus on the elucidation of *in vivo* role of JAG2 in CRC chemoresistance using tissue-specific JAG2 knockout animals.

CRC is one of the leading causes of cancer-related deaths. The development of resistance to chemotherapy has been a significant problem in the successful treatment of cancer. With regards to CRC, the response rate to systemic therapy is 50%, but resistance develops in nearly all patients [50]. Therefore, developing treatment strategies to overcome chemoresistance is imperative. Our study suggests a novel role of JAG2 in tumor resistance to chemotherapy. Since the expression of JAG2 in the normal epithelium is low [21], selective targeting of JAG2 may present a novel therapeutic strategy to sensitize CRC to therapy without affecting normal epithelium.

## MATERIALS AND METHODS

### Materials

JAG1 (#2620), JAG2 (#2210), HES1 (#11988), cleaved caspase-3 (#9661), and GAPDH (#2118) antibodies were purchased from Cell Signaling Technology. p21 (#556430) antibody was purchased from BD Biosciences. Doxorubicin, 5-fluorouracil, oxaliplatin, and DAPT were purchased from Cayman Chemical.

### Animal use

To generate Apc^CKO^: Villin-CreERT^2^ mouse, Apc^CKO^ mice which have floxed Apc alleles were crossed to Villin-CreERT^2^ mice, which expresses tamoxifen-inducible Cre recombinase in the intestinal epithelial cells. The resulting Apc^CKO^:Villin-CreERT^2^ mice were intraperitoneally injected with tamoxifen to induce deletion of Apc gene and sacrificed 4 days later. All animal studies and procedures were approved by the University of South Carolina Institutional Animal Care and Use Committee.

### Cell culture

HCT116, SW480, SW620, LS174T, and HT29 cells were obtained from the American Type Culture Collection (ATCC), and were grown in regular DMEM supplemented with 10% FBS and gentamycin. 1 × 10^6^ cells were plated in a 60 mm cell culture dish and, 48 hours later, cells were treated with doxorubicin or other chemotherapeutic agents in DMSO. p53-null and p21- null HCT116 cells were kindly provided by Dr. Vogelstein.

### Western blot analysis

Cells were washed with cold phosphate-buffered saline (PBS), and lysed on ice in modified RIPA buffer (50 mM Tris–HCl, pH 7.4, 1% NP-40, 0.5% Na-deoxycholate, 0.1% SDS, 1 mM Na3VO4, 10mM NaPPi, 10mM beta-glycerophosphate and 50 mM NaF) supplemented with the Halt protease inhibitor cocktail (Thermo Scientific). Protein concentration was measured using BCA protein assay kit (Pierce). Equal amounts of protein were solubilized and heated at 75°C in LDS sample buffer (Invitrogen) with sample reducing agent (Invitrogen) for 10 min, and then separated by SDS-PAGE, and transferred to Immobilon-P membrane (Millipore). Following incubation in blocking buffer (TBS with 5% nonfat dry milk and 0.1% Tween 20) for 1 hour at room temperature, the membranes were probed overnight at 4°C. The membranes were washed, and then probed with an HRP–linked secondary antibody (Cell Signaling Technology) for 1 hour at room temperature. Specific proteins were detected using an enhanced chemiluminescence (ECL) Western blotting detection kit (GE Healthcare) according to the manufacturer's instructions. All Western blot analysis was repeated for at least three times.

### Immunohistochemistry

Mouse intestinal tissues and grafted tumors were collected, fixed in 10% neutral buffered formalin, and embedded in paraffin blocks. Tissue sections (5μ) were subjected to immunohistochemical analysis Antigen retrieval was performed by immersing sections in Glycine (50mM)-EDTA (0.1% w/v) solution (pH 3.5) at 95°C for 20 min. Sections were incubated with primary antibody [JAG2 (1:500), Santa Cruz Biotechnology; HES1 (1:500), Cell Signaling] at 4°C overnight. Washed sections were incubated in the ImmPress reagent (Vector Laboratories) for 30 min and visualized with diaminobenzidine. After mounting, the sections were observed under an Olympus BX51 light microscope, and the image was acquired by an AxioCam MRc camera.

### Lentiviral vectors

Lentivirus packaging plasmids encoding the packaging proteins Gag-Pol, Rev, Tat and the G protein of the vesicular stomatitis virus were a generous gift from Dr. Gunag Hu (National Institute of Environmental Health Sciences/NIH). The pHAGE-CMV lentivirus backbone was a generous gift from Dr. Darrell Kotton (Boston University School of Medicine). cDNA encoding human p21 was amplified by PCR using primers with added restriction sites and cloned into XbaI and BamHI sites in pHAGE-CMV.

### Viability assay

Cells were seeded in 6-well plates (0.5 × 10^6^ cells/well), and, 48 hours later, treated with doxorubicin in DMSO. At the end of the treatment, both the adherent and detached cells were collected and stained with trypan blue dye for 5 min at room temperature. Cell viability was measured by using the TC10 automated cell counter (Bio-Rad), following manufacturer's instructions. The cells stained with trypan blue were considered as dead cells. For simultaneous observation of viable and non-viable cells, both the adherent and detached cells were collected and incubated in PBS containing Calcein AM (0.2 μM) and DAPI (1 μg/ml) for 10 min at 37°C. The number of live and dead cells was counted under a fluorescent microscope and the percentage of live cells was calculated as cell viability.

### RT-PCR and real-time PCR

RNA was isolated using an RNeasy kit (Qiagen) and treated with 1 unit of amplification grade DNase I (Invitrogen) per 1 μg RNA at room temperature for 15 min to remove genomic DNA followed by inactivation of the DNase I with 2.5 mM EDTA (pH 8.0) and incubation at 65°C for 5 min. Reverse transcription was done with 2 μg total RNA using SuperScript II reverse transcription system (Invitrogen) according to the manufacturer's instructions. For RT-PCR analysis, an initial amplification was done with a denaturation step at 95°C for 3 min, followed by denaturation at 95°C for 30 seconds, primer annealing at 58°C for 30 seconds, and primer extension at 72°C for 30 seconds. The primer sequences are provided in Supplementary Table 1. Upon completion of the cycling steps, a final extension was performed at 72°C for 7 min. Quantitative real-time PCR analysis was conducted using a GoTaq® qPCR mixture (Promega). Reactions were run in triplicate for three independent experiments. The mean of housekeeping gene GAPDH was used as an internal control to normalize the variability in expression levels. The primer sequences for real-time PCR analysis are provided in Supplementary Table 2. Expression data were normalized to the mean of GAPDH to control the variability in expression levels and were analyzed using the 2^−ΔΔCT^ method.

### Establishment of stable knockdown and over-expressing cell lines by lentiviral vector

The HEK 293T/17 cell line (ATCC) was used as a packaging cell line and was transfected with lentiviral packaging vectors and a target vector. To generate JAG1 or JAG2-knockdown cell lines, target shRNA sequences were designed by web-based shRNA sequence designing software (http://www.broadinstitute.org/rnai/public/seq/search). The sequences of JAG1 and JAG2 shRNA are provided in Supplementary Table 3 of Supplementary information. For the ectopic expression, human and mouse JAG2 cDNAs were cloned into a pHAGE-CMV vector. The viral particles were harvested 48 hours later and were then incubated with the cell lines for the next 24 hours in the presence of polybrene (8 μg/ml). A shRNA lentiviral vector (pLBIP) contains EGFP and a puromycin resistance gene. The efficiency of lentiviral infection was assessed by EGFP fluorescence under a microscope. The transduced cells were selected and maintained in puromycin (2 μg/ml)-containing media.

### Cell cycle analysis

Cells were fixed and stained as previously described [51]. Briefly, 1×10^6^ cells were seeded in 60-mm dishes. After 48 hours, the cells were treated with 1 μM doxorubicin or vehicle for 48 hours. The attached cells were then trypsinized and combined with the floating cells. The cells were resuspended in PBS, fixed with ice-cold 70% ethanol for ≥20 min and stored at −20°C. Fixed cells were rinsed again and re-suspended in PBS containing 50 μg/ml propidium iodide (PI) (Sigma) and 100 μg/ml DNase-free RNase (Sigma). The cells were then run on a Becton-Dickinson FACScan flow cytometer (BD Biosciences).

### Xenograft tumorigenesis in nude mice

All animal procedures were approved by the Animal Care and Use Committee of the University of South Carolina. Athymic nude mice (BALB/c nu/nu) (female, 6 week-old) were purchased from the Jackson Laboratory, and were acclimated for 7 days in the laboratory before experimentation. To establish xenograft tumors, 1 × 10^6^ JAG2-knockdown and control HCT116 cells or 1 × 10^6^ control and JAG2 over-expressing MC38 cells in Matrigel^®^ were injected subcutaneously into the dorsal flanks of the mice as described [52]. Tumor volumes were calculated according to the following formula: volume (mm^3^) = [(shortest diameter)^2^ × longest diameter]/2. On the day of harvest, the tumor tissues were excised for measurement of tumor size.

### Statistical analysis

Data were expressed as the mean ± SD (standard deviation) of at least three independent experiments. The statistical analysis was done using a Student's t-test or One-way ANOVA, using GraphPad Prism 5 software (GraphPad Software). Statistical significance is indicated by asterisks in figures: * for p values <0.05, ** for p values <0.01, and *** for p values <0.001.

## SUPPLEMENTARY FIGURES AND TABLES


