# Supplementary material for: Jagged-2 (JAG2) enhances tumorigenicity and chemoresistance of colorectal cancer cells
Source: Oncotarget. 2017 Jun 7;8(32):53262–75. doi: 10.18632/oncotarget.18391 (PMC5581108; doi:10.18632/oncotarget.18391)
Supplement: Supplementary file 1 [file oncotarget-08-53262-s001.pdf]

Jagged-2 (JAG2) enhances tumorigenicity and chemoresistance of colorectal cancer cells

SUPPLEMENTARY MATERIALS

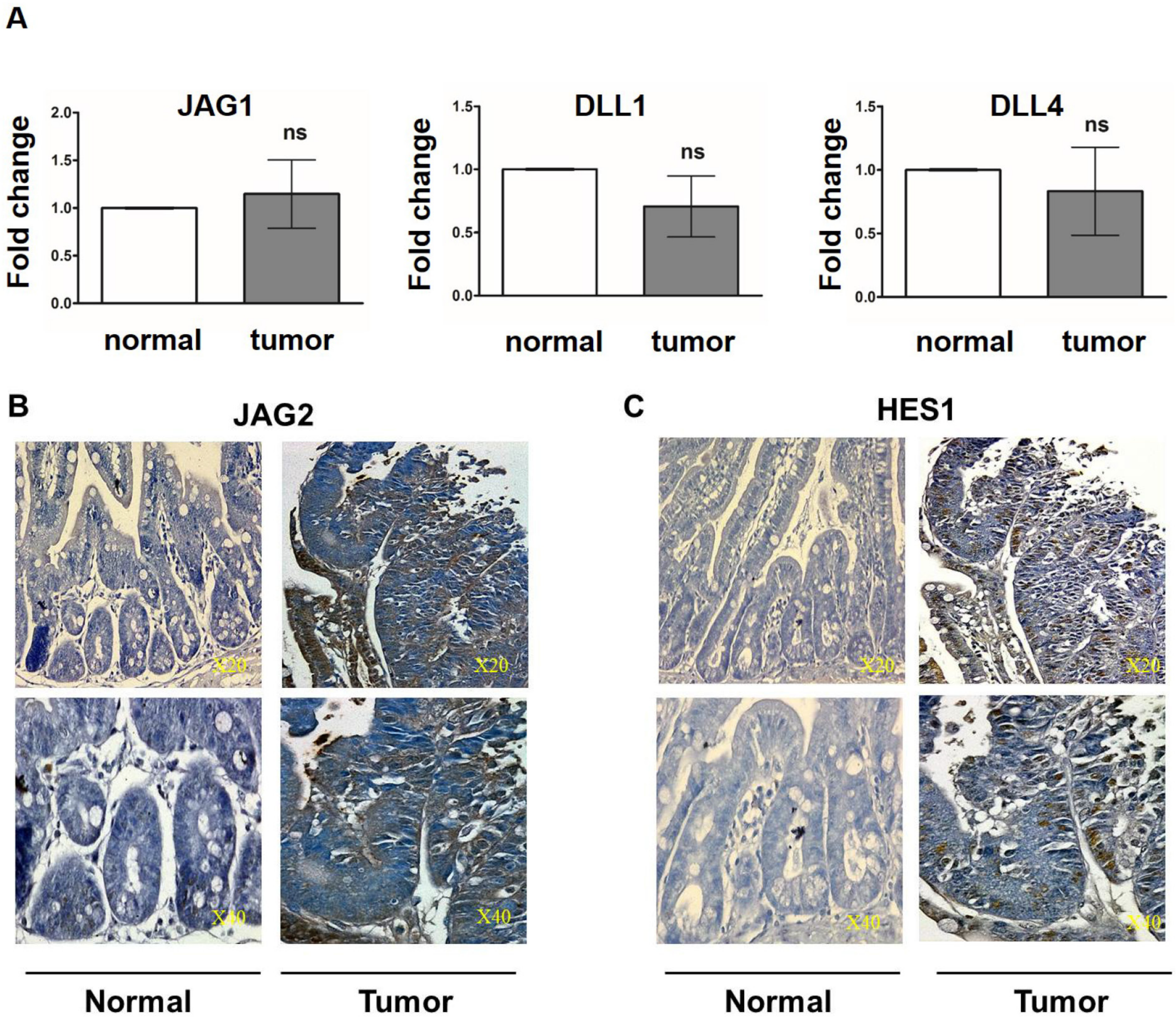

Supplementary Figure 1: (A) Quantitative real-time PCR (qRT-PCR) analysis for JAG1, DLL1, and DLL4 expression in normal and tumor samples of *APC*$^{Min/+}$ mice. The expression of DLL3 was undetectable. NOTCH ligand expression in 5 tumor samples was calculated as mean fold change relative to surrounding normal colon using the $2^{-\Delta\Delta C_T}$ method (ns: non-significant). **(B)** Immunohistochemistry of JAG2 and **(C)** HES1 in tumors of Apc$^{Min/+}$ mice and nearby normal regions.

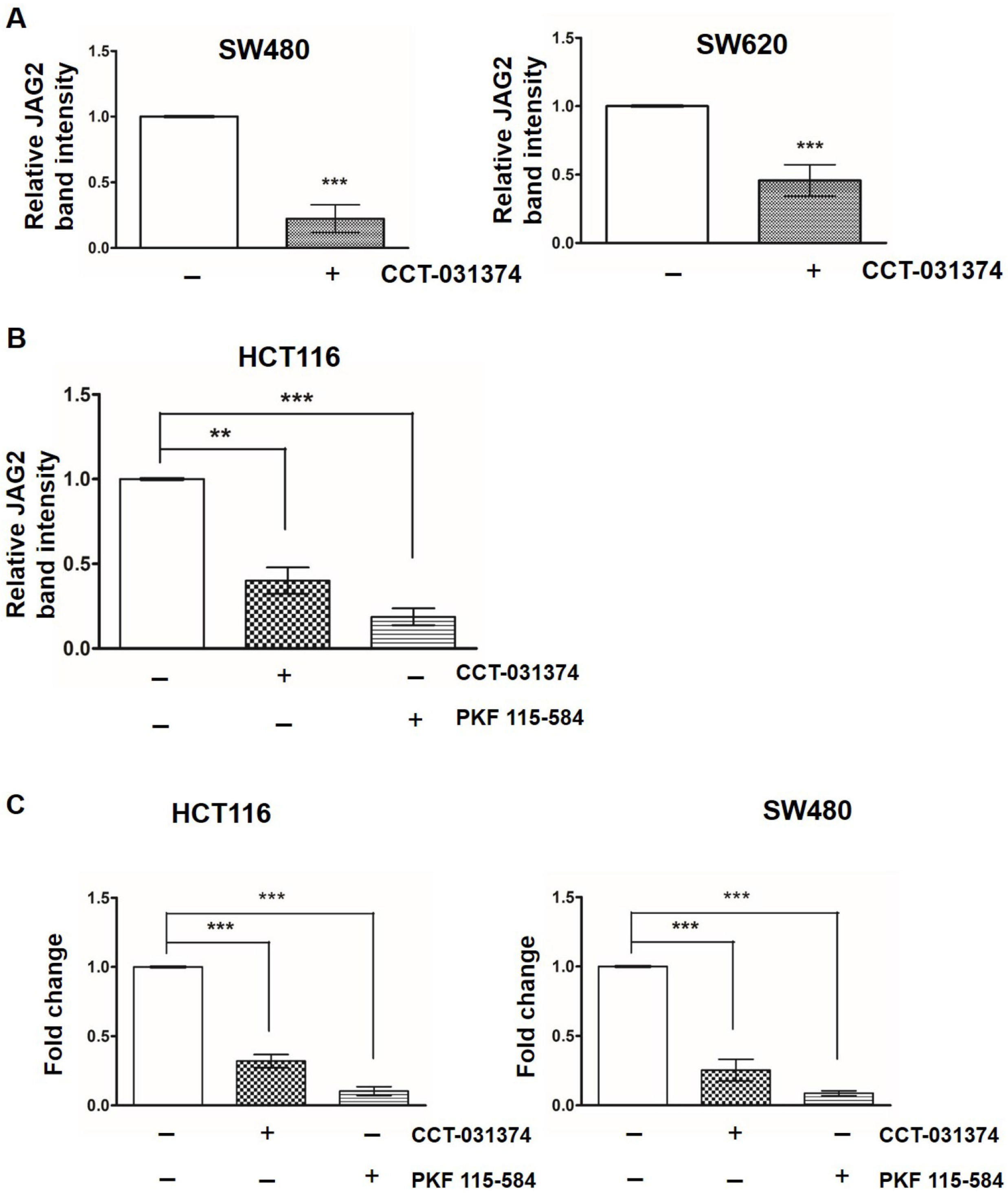

Supplementary Figure 2: (A) The effect of CCT-031374 on JAG2 protein levels in SW480 and SW620 cells. **(B)** The effect of β-catenin signaling inhibitors on JAG2 protein levels in HCT116 cells. The graph shows fold changes of GAPDH-normalized JAG2 protein levels over vehicle-treated control. The band intensities were determined from more than 3 independent Western blots. **(C)** qRT-PCR analysis for JAG2 expression in HCT116 and SW480 cells treated with CCT-031374 or PKF 115-584. The cells were incubated with 1 μM PKF 115-584 or 25 μM CCT-031374 for 24 hours. JAG2 expression was calculated as mean fold change relative to vehicle-treated cells.

(*Continued*)

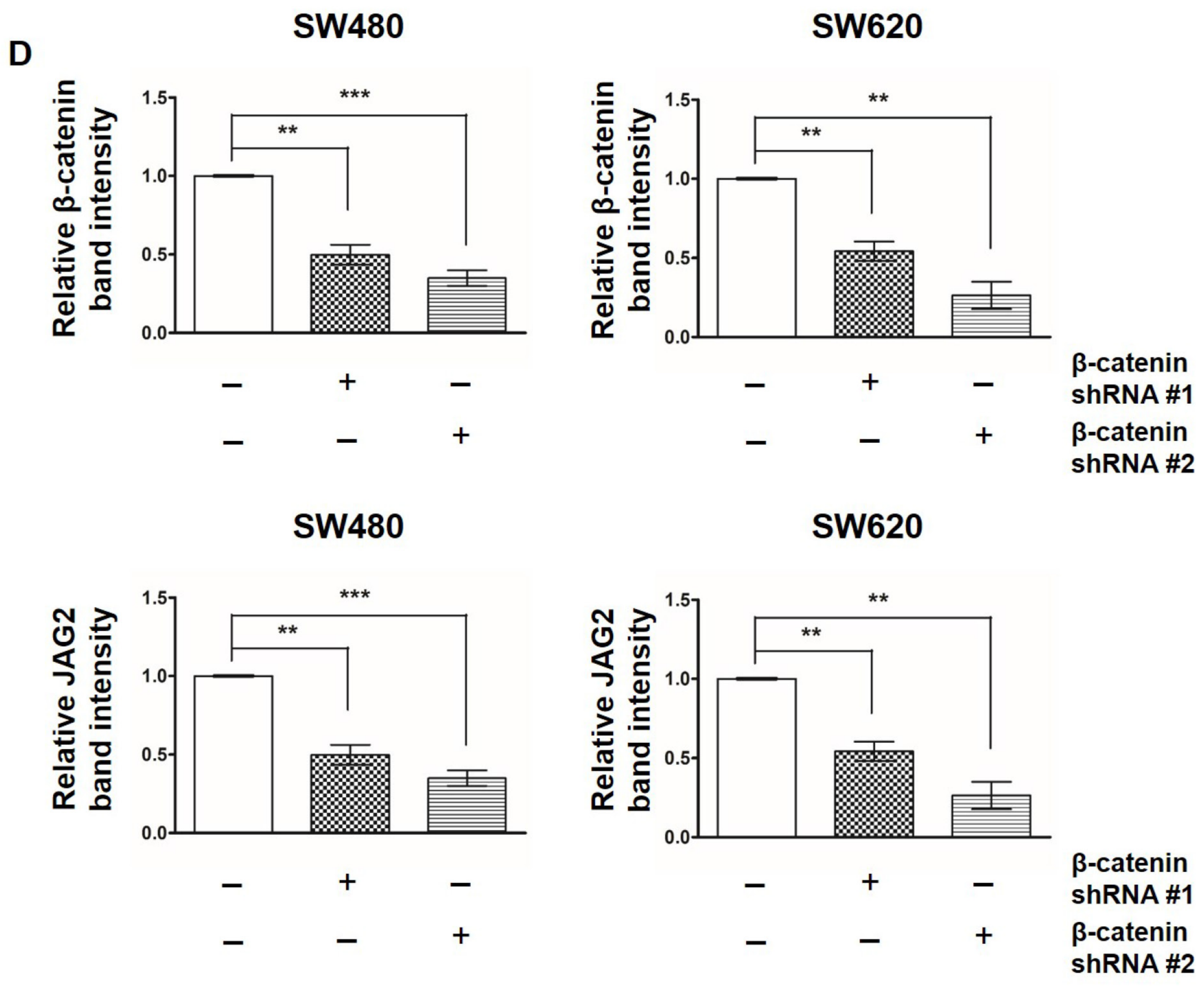

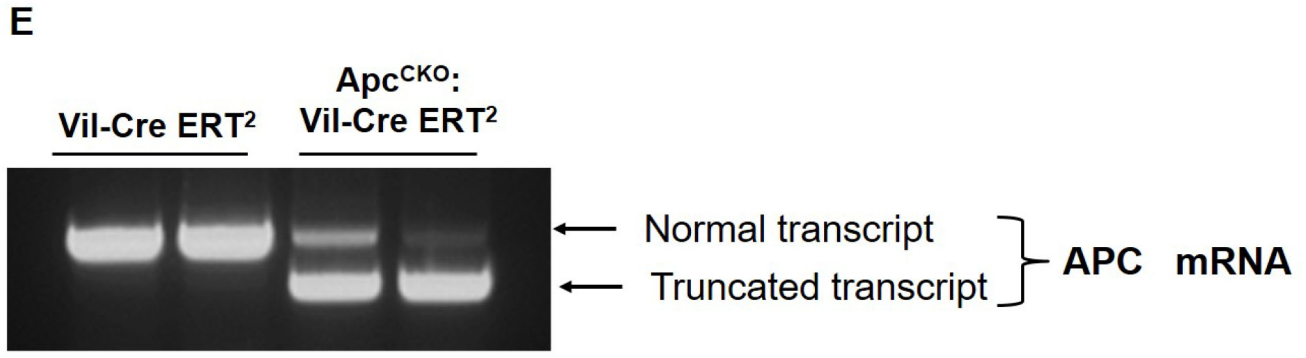

Supplementary Figure 2 (*Continued*): (D) The effect of β-catenin knockdown on JAG2 protein levels in SW480 and SW620 cells. The graph shows fold changes of GAPDH-normalized JAG2 protein levels over empty vector-transduced control. The band intensities were determined from more than 3 independent Western blots. **(E)** RT-PCR analysis of Apc mRNA isolated from the intestine of tamoxifen-induced Vil-CreERT^2 and Apc^{CKO}:Vil-CreERT^2 mice. Truncated mRNA transcripts of Apc gene were observed in the intestines of tamoxifen-induced Apc^{CKO}:Vil-CreERT^2 mice as compared to normal transcripts in the intestines of Vil-CreERT^2 mice.

(*Continued*)

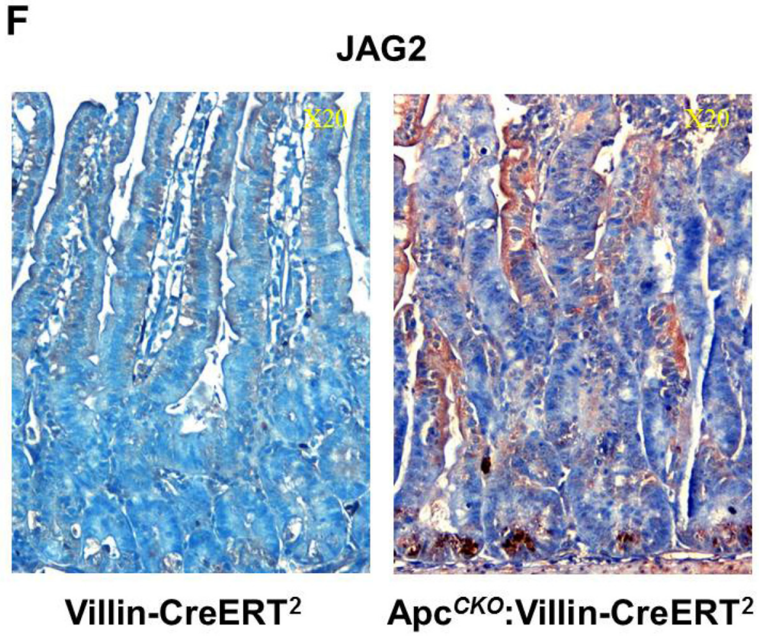

Supplementary Figure 2 (*Continued*): (F) Immunohistochemistry of JAG2 in Villin-CreERT^{2} and Apc^{CKO}**:**Villin-CreETR^{2} mice intestines after 4 days of tamoxifen treatment. Deletion of Apc allele due to tamoxifen-induced Cre expression leads to the expression of JAG2 in Apc^{CKO}:Villin-CreETR^{2} mice intestines.

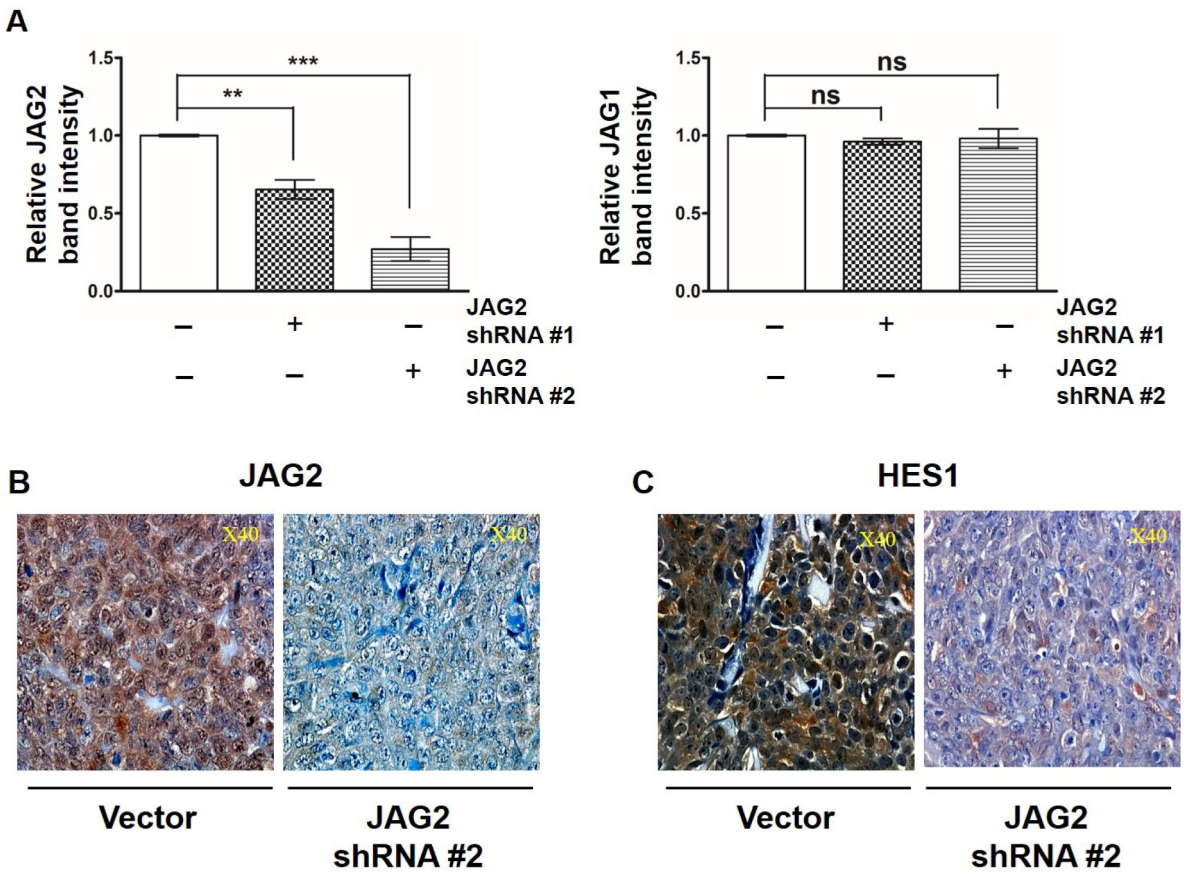

Supplementary Figure 3: (A) The levels of JAG2 and JAG1 protein in stable JAG2 shRNA-expressing HCT116 cells. The graph shows fold changes of GAPDH-normalized JAG2 and JAG1 protein levels over empty vector-transduced control. The band intensities were determined from more than 3 independent Western blots. Immunohistochemistry of JAG2 **(B)** and HES1 **(C)** in subcutaneous tumors of empty vector and JAG2 shRNA-expressing HCT116 cells. HCT116 control cell-derived tumors have higher levels of JAG2 and HES1 as compared to JAG2 shRNA-expressing tumors.

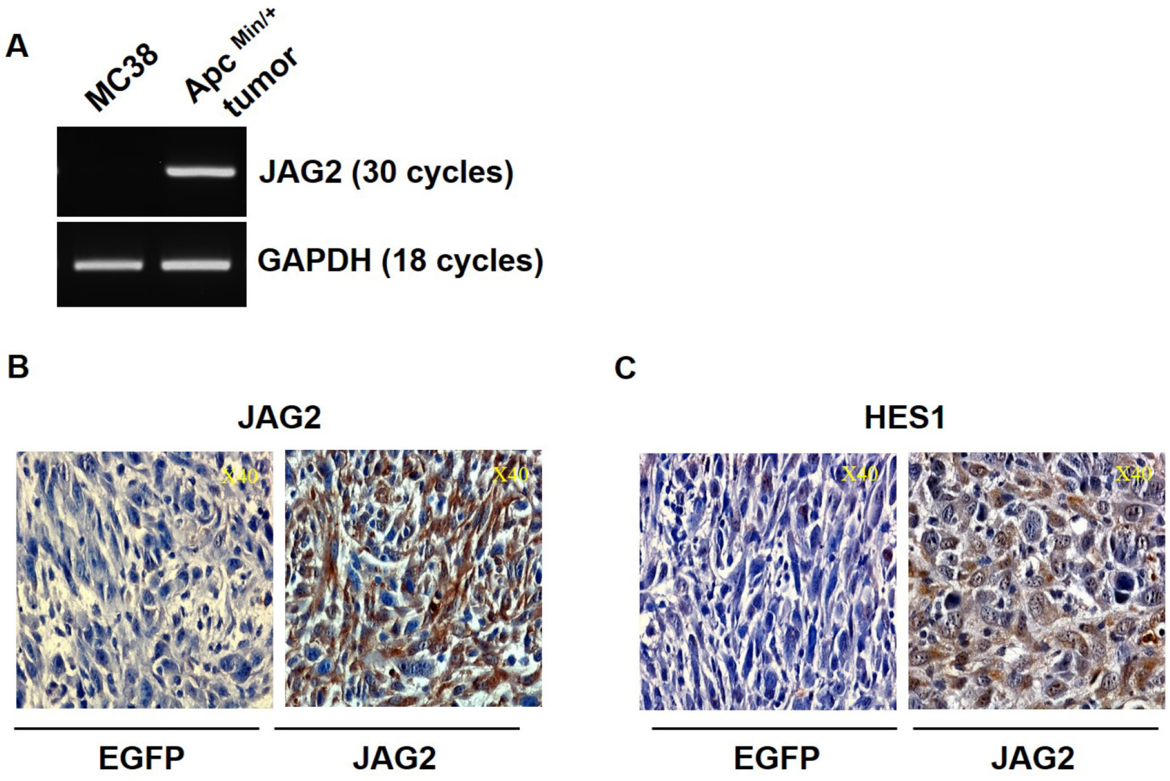

Supplementary Figure 4: **(A)** RT-PCR analysis for JAG2 in MC38 cells. RNA from *APC*$^{Min/+}$ tumor was used as a positive control. GAPDH served as a loading control. Immunohistochemistry of JAG2 **(B)** and HES1 **(C)** in C57BL/6 mice subcutaneous tumors generated from control and JAG2 over-expressing MC38 cells.

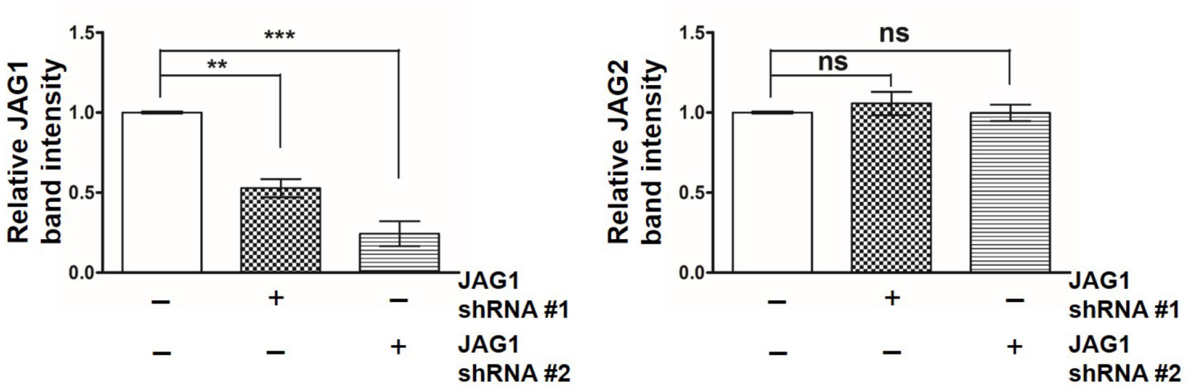

Supplementary Figure 5: The levels of JAG1 and JAG2 protein in stable JAG1 shRNA-expressing HCT116 cells. The graph shows fold changes of GAPDH-normalized JAG1 and JAG2 protein levels over empty vector-transduced control. The band intensities were determined from more than 3 independent Western blots.

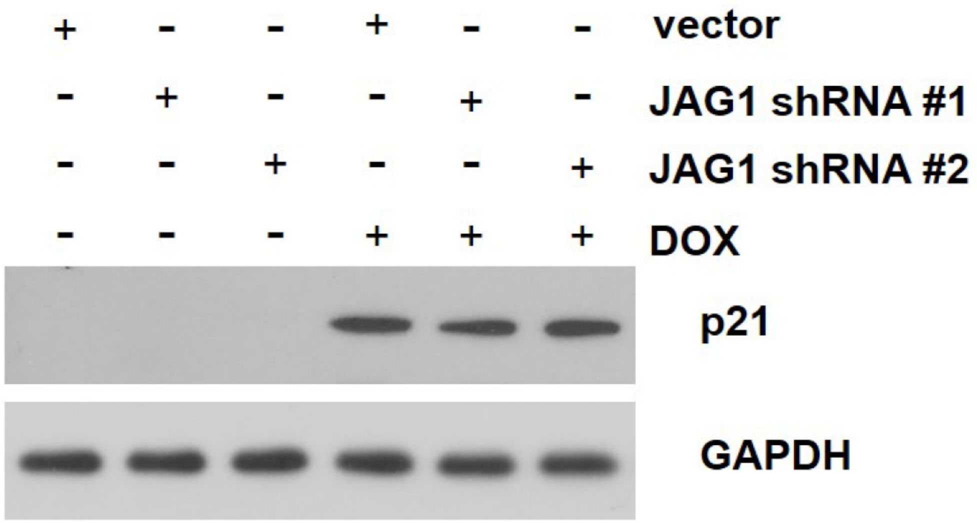

Supplementary Figure 6: Control and JAG1-knockdown HCT116 cells were treated with 1 μM doxorubicin (DOX) for 48 hours. Total cell lysates were subjected to Western blot analysis for p21.

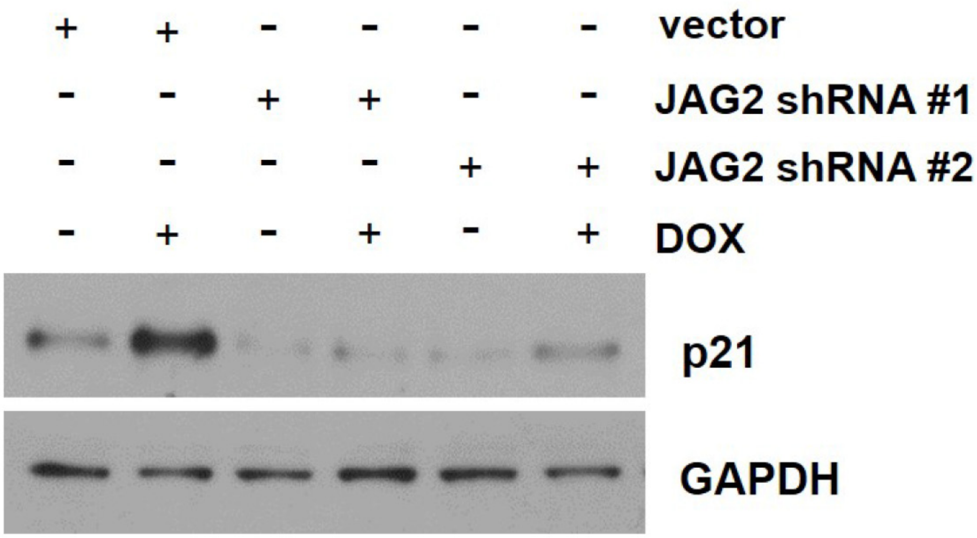

Supplementary Figure 7: Control and JAG2-knockdown p53-null HCT116 cells were treated with 1 µM doxorubicin (DOX) for 48 hours. Total cell lysates were subjected to Western blot analysis for p21.

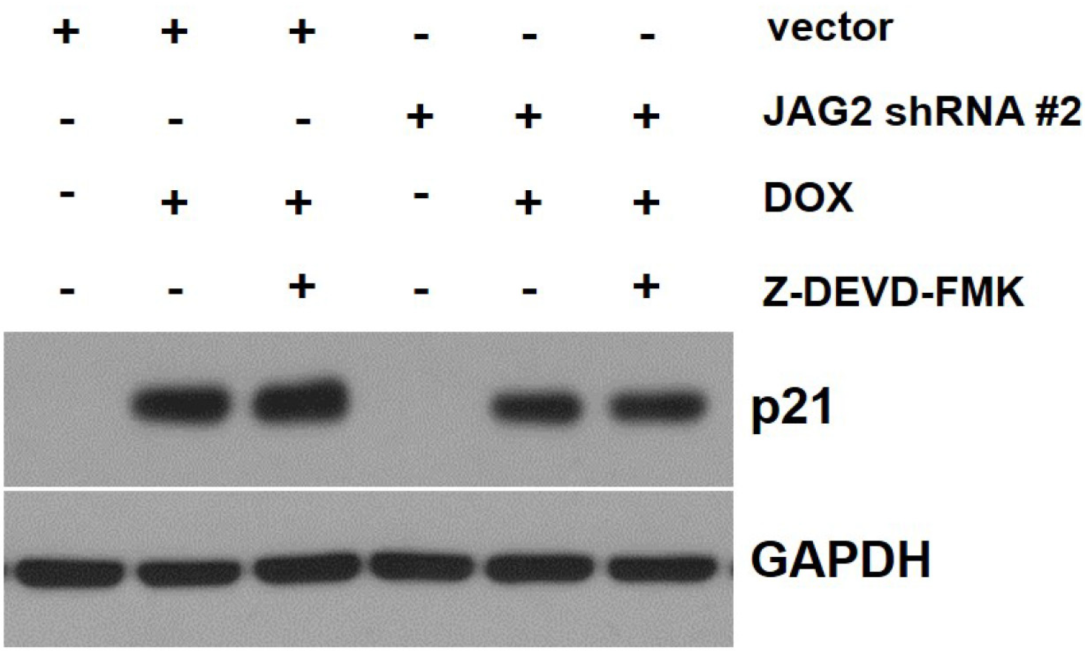

Supplementary Figure 8: Control and JAG2-knockdown HCT116 cells were pre-treated with 30 µM Z-DEVD-FMK for 1 hour and then co-treated with 1 µM doxorubicin (DOX) for 48 hours. Total cell lysates were subjected to Western blot analysis for p21.

Supplementary Table 1: The general PCR primer sequences

Name of target gene	Forward primer sequence	Reverse primer sequence	Target product size
**mJAG1**	5′- GGCCAAACCTTGTGTAAATG -3′	5′-GAGCAGTTCTTGCCCTCATA-3	432
**mJAG2**	5′-TGCCAGGAAGTGGTCATATT -3	5′-TCGGATTCCAGAGCAGATAG -3′	411
**mDLL1**	5′-ATGGTCTCAGGACCTTCACA -3′	5′-AGTTCAGGTCTTGGTTGCAG -3′	398
**mDLL3**	5′-CTTCTCCCTCGTCATTGAAA -3′	5′-AGTTCAGGCAGCTACTGGTG -3′	422
**mDLL4**	5′-AAAATGACACCCTCACCAGA -3′	5′-GTATAACCCTTTGGCCCACT -3′	448
**mGAPDH**	5′-TCAGGAGAGTGTTTCCTCGT-3′	5′-GCGGAGATGATGACCCTTTT-3′	394

Supplementary Table 2: The real-time PCR primer sequences

Name of target gene	Forward primer sequence	Reverse primer sequence	Target product size
**mJAG2**	5′-ACATCAACGATTGCCATGGG-3′	5′-TGGCACACTTGTCGTACTCT-3′	129
**mGAPDH**	5′-GGACCTCATGGCCTACATGG-3′	5′-TAGGGCCTCTCTTGCTCAGT-3′	85
**hJAG2**	5′-TACCAACGACTGCAACCCTC-3′	5′-GCACTCGTCGATGTTGATGC-3′	127
**hp21**	5′-TCTTGTACCCTTGTGCCTCG-3′	5′-ATCTGTCATGCTGGTCTGCC-3′	102
**hGAPDH**	5′-CAATGACCCCTTCATTGACC-3′	5′-GACAAGCTTCCCGTTCTCAG-3′	106

Supplementary Table 3: The sequences of shRNA oligomers

Target gene		Sequences
**JAG1 shRNA #1**	**Sense**	5′- TGAATGTTTCCGCTGAATATTTCAAGAGAATATTCAGCGGAAACATTCTTTTTTC -3′
	**Anti-sense**	5′- TCGAGAAAAAAGAATGTTTCCGCTGAATATTCTCTTGAAATATTCAGCGGAAACATTCA -3′
**JAG1 shRNA #2**	**Sense**	5′- TGCTTAAACCGAATGGAGTATTCAAGAGATACTCCATTCGGTTTAAGCTTTTTTC -3′
	**Anti-sense**	5′- TCGAGAAAAAAGCTTAAACCGAATGGAGTATCTCTTGAATACTCCATTCGGTTTAAGCA -3′
**JAG2 shRNA #1**	**Sense**	5′- TGGAAGCTGTGTGTAAACAATTCAAGAGATTGTTTACACACAGCTTCCTTTTTTC -3′
	**Anti-sense**	5′- TCGAGAAAAAAGGAAGCTGTGTGTAAACAATCTCTTGAATTGTTTACACACAGCTTCCA -3′
**JAG2 shRNA #2**	**Sense**	5′- TGGACTAAATACCATTCCATTTCAAGAGAATGGAATGGTATTTAGTCCTTTTTTC -3′
	**Anti-sense**	5′- TCGAGAAAAAAGGACTAAATACCATTCCATTCTCTTGAAATGGAATGGTATTTAGTCCA -3′
**β-catenin shRNA #1**	**Sense**	5′-TGGCTGCAGTTATGGTCCATTTCAAGAGAATGGACCATAACTGCAGCCTTTTTTC-3′
	**Anti-sense**	5′-TCGAGAAAAAAGGCTGCAGTTATGGTCCATTCTCTTGAAATGGACCATAACTGCAGCCA-3′
**β-catenin**	**Sense**	5′- TCGAGAAAAAAGGACTAAATACCATTCCATTCTCTTGAAATGGAATGGTATTTAGTCCA -3′
**shRNA #2**	**Anti-sense**	5′- TGGACTAAATACCATTCCATTTCAAGAGAATGGAATGGTATTTAGTCCTTTTTTC -3′
